# Supplementary material for: Diversity and Multiple Infections of Bartonella in Red Deer and Deer Keds
Source: Pathogens. 2024 Dec 27;14(1):6. doi: 10.3390/pathogens14010006 (PMC11768248; doi:10.3390/pathogens14010006)
Supplement: Supplementary file 1 [file pathogens-14-00006-s001.zip › pathogens-3372542-supplementary.pdf]

pathogens

MDPI

Supplementary Materials

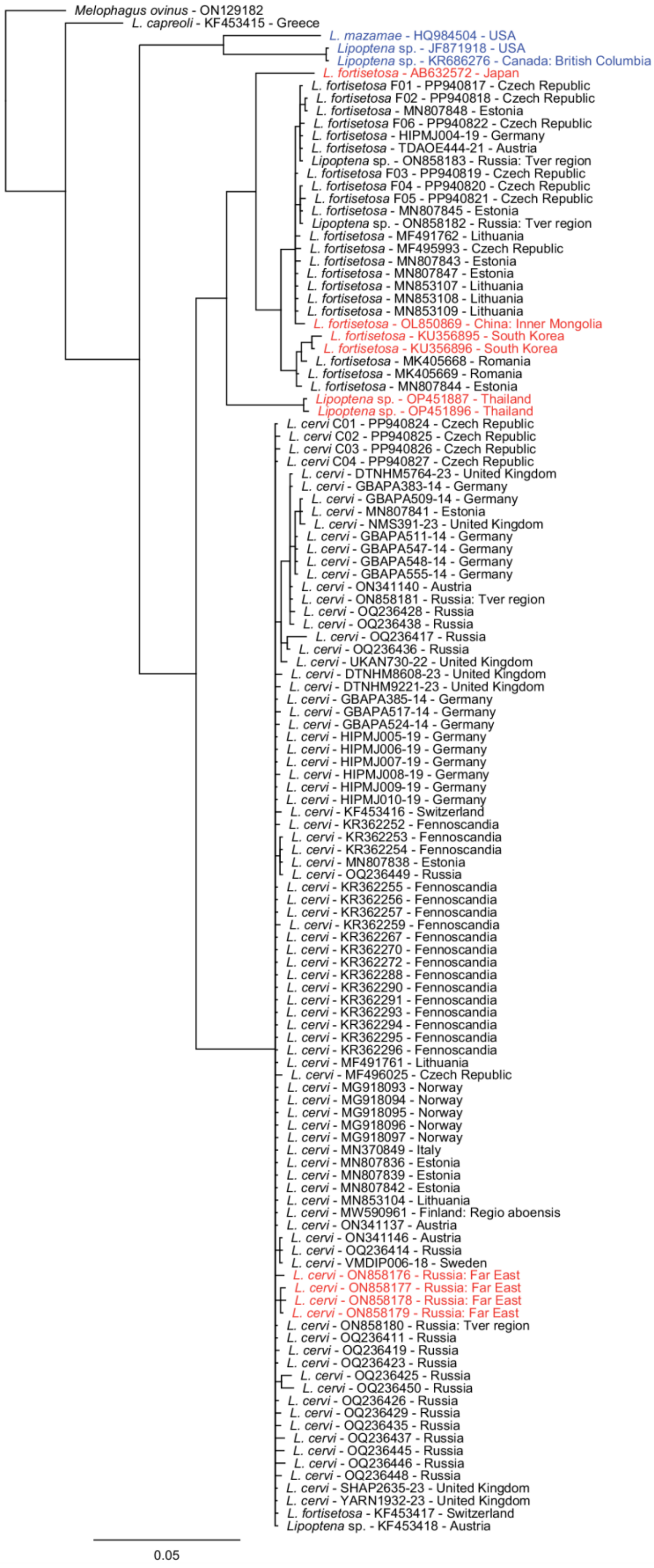

 https://doi.org/10.3390/xxxxx

Figure S1. Phylogenetic tree of *Lipoptena* COI sequences from this study (C01-C04, F01-F06), Genbank and BOLD. Isolates from Europe are in black, isolates from Asia in red and isolates from America in blue. The tree was constructed with Bayesian inference.

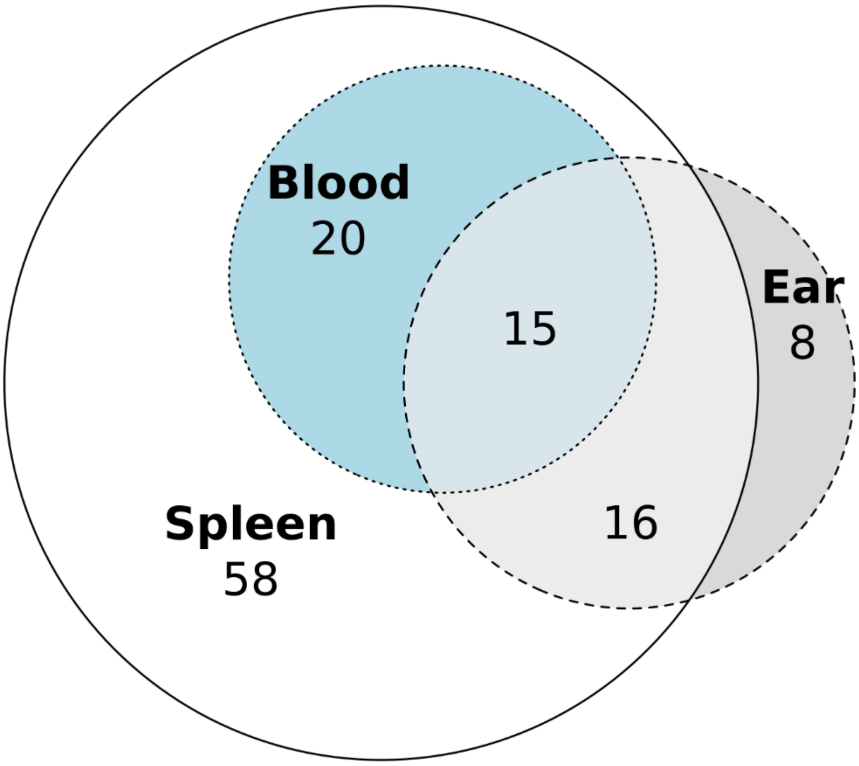

Figure S2. Percentage of positive tissue samples in *Bartonella*-positive deer. For blood samples, only deer with available blood samples were included in the calculation, with each duplicate counted as a separate sample.

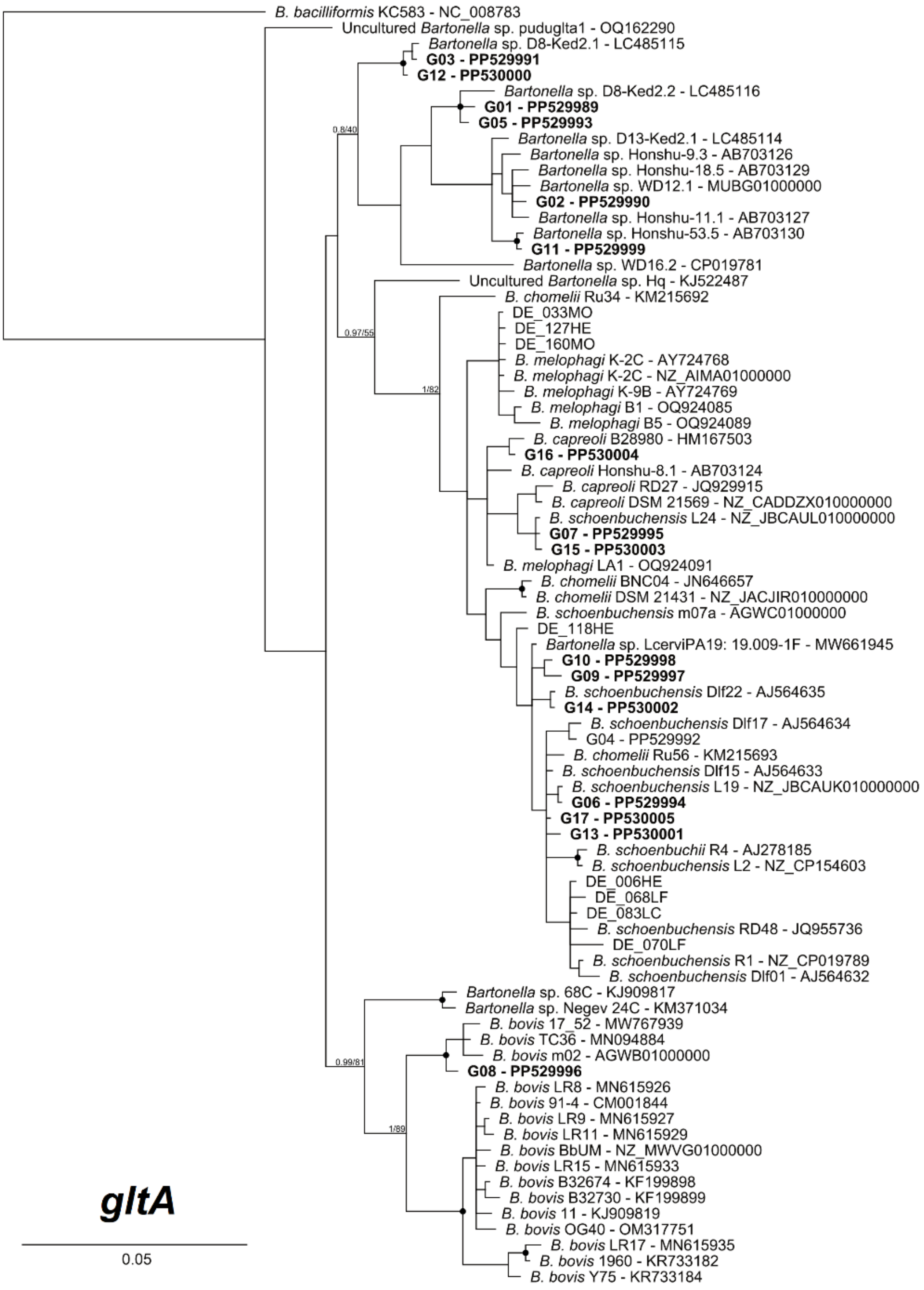

Figure S3. Phylogenetic tree of sequences at the *gltA* locus, featuring genotypes G01-G17, 55 unique sequences from Genbank and 7 sequences from [44]. Bayesian inference was used for tree construction, with support values calculated using Maximum Likelihood with 500 bootstraps. Nodes with robust support (near 100%) in both models are indicated by black dots. Selected nodes are marked with numbers indicating Bayesian support/bootstrap support.

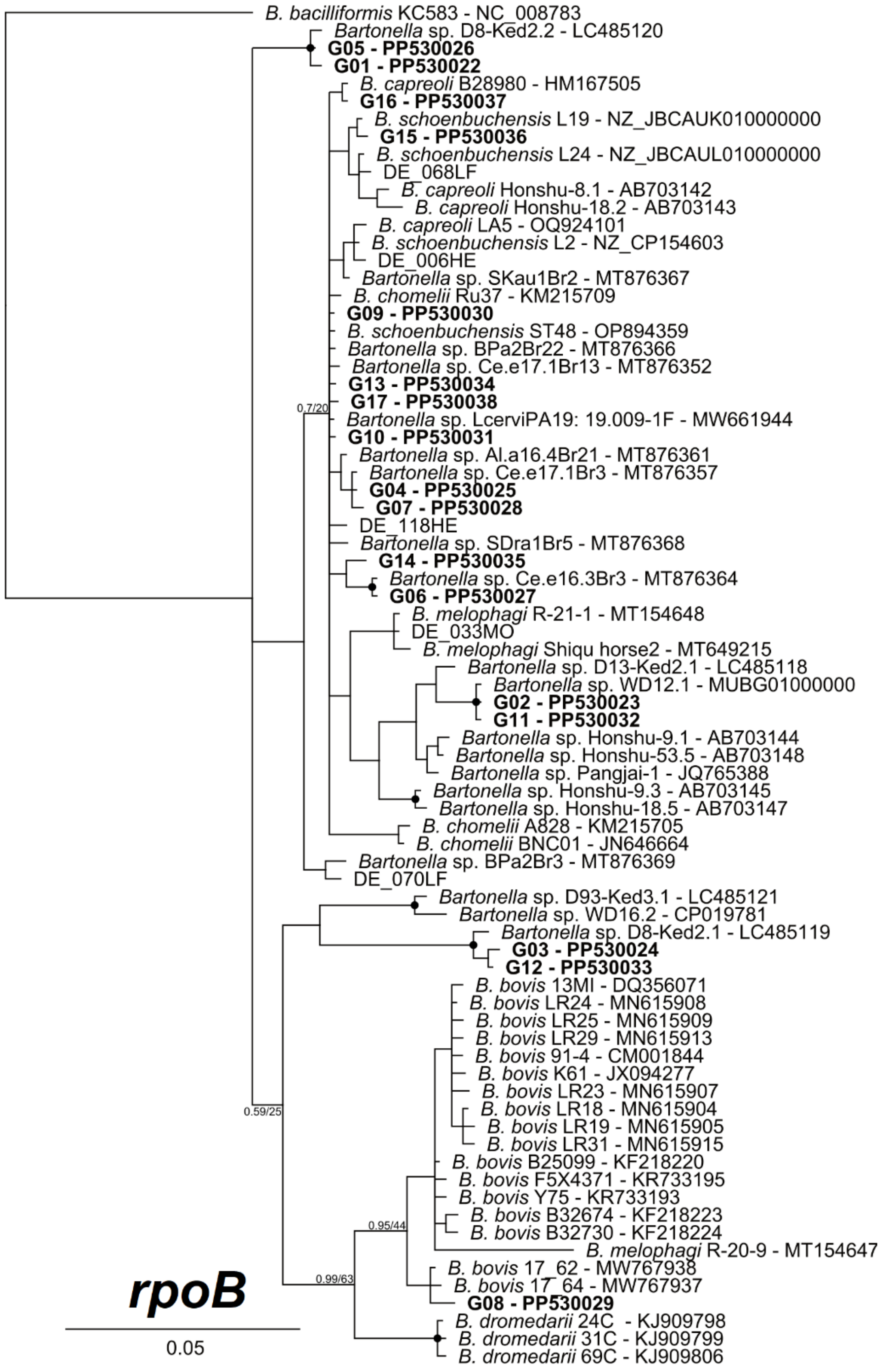

Figure S4. Phylogenetic tree of sequences at the *rpoB* locus, featuring genotypes G01-G17, 54 unique sequences from Genbank and 5 sequences from [44]. Bayesian inference was used for tree construction, with support values calculated using Maximum Likelihood with 500 bootstraps. Nodes with robust support (near 100%) in both models are indicated by black dots. Selected nodes are marked with numbers indicating Bayesian support/bootstrap support.

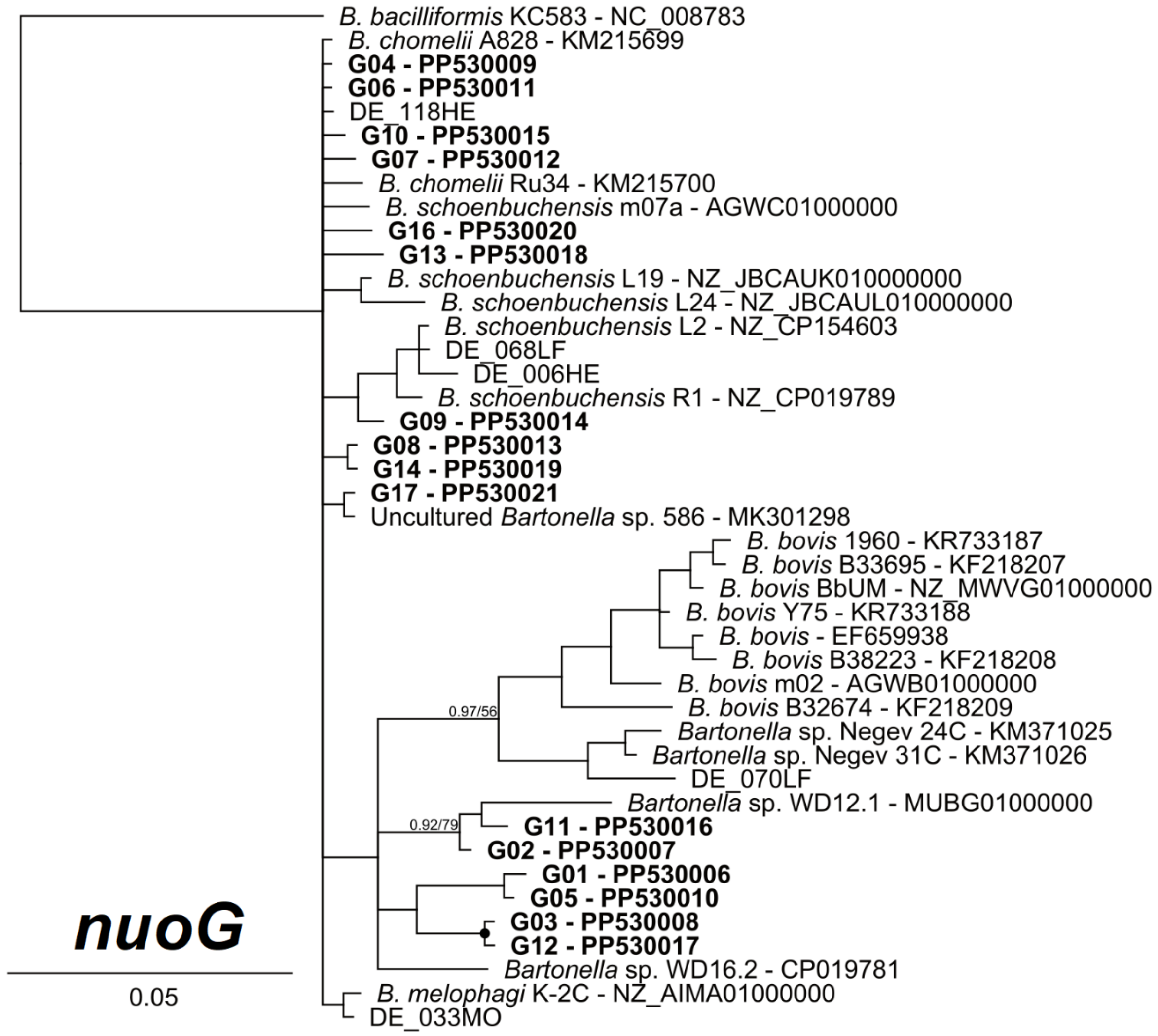

Figure S5. Phylogenetic tree of sequences at the *nuoG* locus, featuring genotypes G01-G14, G16 and G17, 21 unique sequences from Genbank and 5 sequences from [44]. Bayesian inference was used for tree construction, with support values calculated using Maximum Likelihood with 500 bootstraps. Nodes with robust support (near 100%) in both models are indicated by black dots. Selected nodes are marked with numbers indicating Bayesian support/bootstrap support.

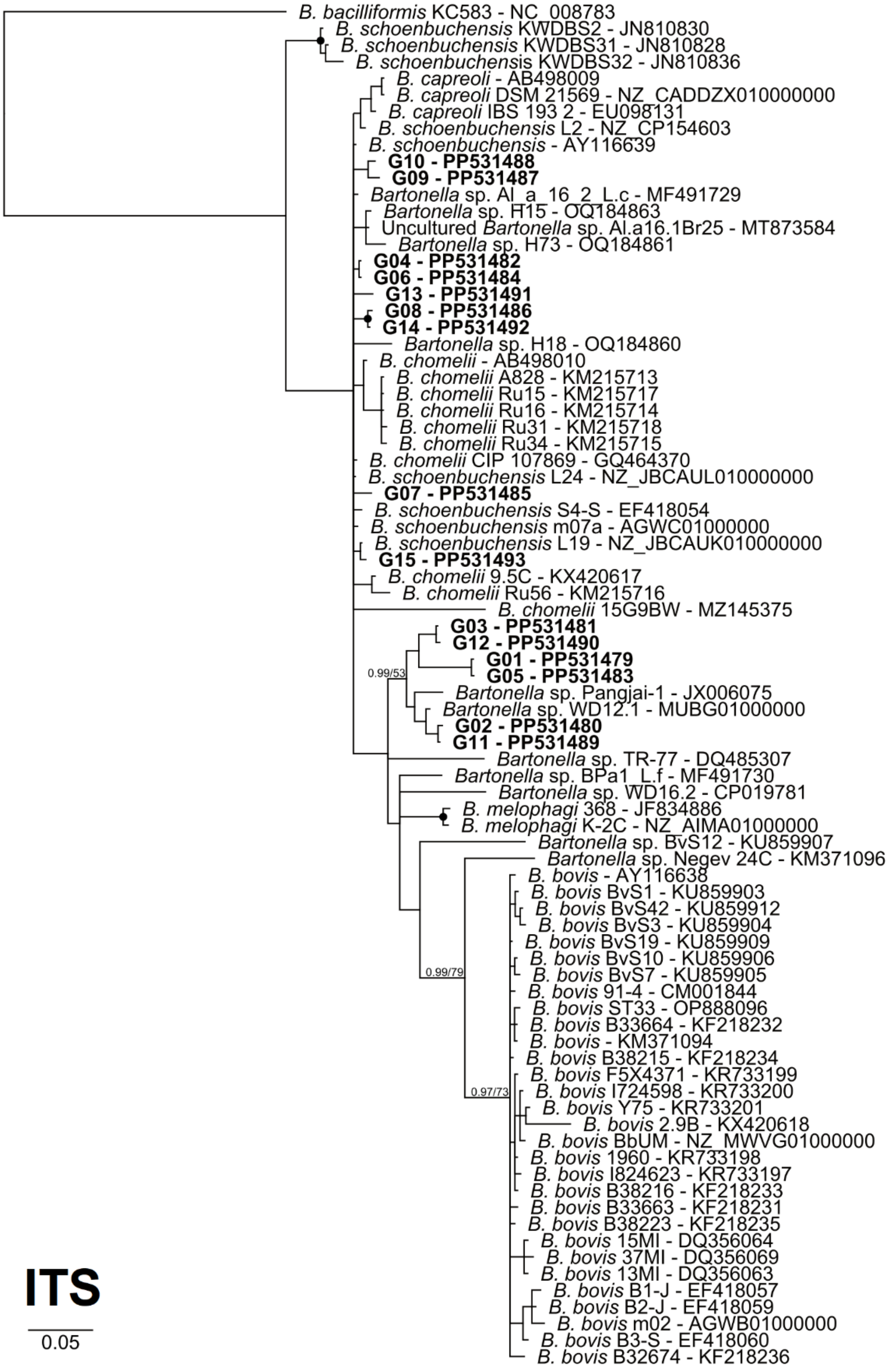

Figure S6. Phylogenetic tree of sequences at the ITS locus, featuring genotypes G01-G15 and 66 unique sequences from Genbank. Bayesian inference was used for tree construction, with support values calculated using Maximum Likelihood with 500 bootstraps. Nodes with robust support (near 100%) in both models are indicated by black dots. Selected nodes are marked with numbers indicating Bayesian support/bootstrap support.
